# Accuracy of LexisNexis-derived retrospective address histories in the Sister Study cohort

**DOI:** 10.1038/s41370-025-00802-1

**Published:** 2025-08-25

**Authors:** Jennifer L. Ish, Meklit Daniel, Patrick Ringwald, Nicole M. Niehoff, Rena R. Jones, Alexandra J. White

**Affiliations:** 1 Epidemiology Branch, Division of Intramural Research and Training, National Institute of Environmental Health Sciences, Research Triangle Park, NC USA; 2 Westat, Rockville, MD USA; 3 Real-World Evidence & Health Outcomes Research Group, GSK plc, Durham, NC USA; 4 Occupational & Environmental Epidemiology Branch, Division of Cancer Epidemiology & Genetics, National Cancer Institute, Rockville, MD USA

**Keywords:** Residence history, Epidemiology, Exposure assessment, Geospatial analysis

## Abstract

**Background:**

Commercial address data can help reconstruct detailed residential histories, which are crucial for accurate assessment of geospatial-based environmental exposures in epidemiologic studies.

**Objective:**

To reconstruct and assess the accuracy of pre-baseline residential histories for the Sister Study, an ongoing United States-wide prospective cohort.

**Methods:**

We used LexisNexis® Accruint® to construct pre-baseline residential histories for 47,557 participants. A subset (*N* = 823) validated their LexisNexis-derived addresses via a supplemental questionnaire. We assessed the proportion of addresses with verified locations and timeframes by sociodemographic and geographic characteristics.

**Results:**

Residential histories were reconstructed for 93.5% of participants, adding a median of 25 years of data. The histories accurately captured 95% of address locations and 82% of residence durations, with improved accuracy after 1990.

**Impact:**

- This study leverages LexisNexis to reconstruct detailed residential histories before cohort enrollment for nearly all Sister Study participants, creating a valuable resource for investigating the health effects of past environmental exposures. A subset of participants verified the locations and timeframes of a high proportion of addresses in the LexisNexis-derived histories, reinforcing confidence in their accuracy for the full cohort.

## Introduction

Environmental epidemiologic studies commonly estimate exposures by linking geospatial-based exposure information to participants’ residential locations. In the absence of detailed residential histories, which can be burdensome or costly to collect, researchers often rely on the address at study enrollment. However, reliance on exposure at enrollment or another single time point as a proxy for long-term exposure can result in misclassification due to residential mobility [1, 2]. When exposure misclassification is non-differential, effect estimates can be underestimated, making it difficult to identify associations. Further, exposure assessment at the time of study entry may not capture the most etiologically relevant period for diseases with a long latency and precludes the evaluation of delayed effects of exposures during potentially sensitive periods.

Commercial databases that compile public records data have proven to be a useful source of residential history information for epidemiologic studies [3–5]. The commercial database LexisNexis® Accurint® provides address information that covers multiple decades and has been shown to correspond well with available study addresses [6–12]. Here, we used LexisNexis to reconstruct residential histories for participants in the Sister Study, supplementing existing study address data with complete, longitudinal address information prior to enrollment. We described the accuracy of LexisNexis-derived residential histories among a subset of participants and evaluated whether the accuracy varied by sociodemographic characteristics, across geographic regions, and over time.

## Methods

### Study population

The Sister Study is an ongoing prospective cohort study designed to investigate environmental risk factors for breast cancer [13]. Between 2003 and 2009, a total of 50,884 women across the United States and Puerto Rico were enrolled into the study. All participants provided written informed consent prior to enrollment. The collection of data in the Sister Study and linkage with commercial data sources has been approved by the institutional review board of the National Institute of Environmental Health Sciences. We excluded participants who had withdrawn from the study (*n* = 9; Data Release 11.1).

### Sources of address data

LexisNexis is a commercial vendor of data products that aggregates information from public records including credit reporting data, real estate and tax records, property deed transfers and mortgages, driver’s license records, court filings, and state death registries. We requested up to 20 most recent addresses for all Sister Study participants and provided LexisNexis with the following information: full names, gender, date of birth, enrollment address, phone number, and date of death (when applicable). We also utilized the United States Postal Service (USPS) Residential Delivery Indicator product (RDI; https://postalpro.usps.com/address-quality-solutions/residential-delivery-indicator-rdi) to identify business addresses. Self-reported residence history at baseline included the street address and dates of residence of participants’ primary residence at study enrollment and where they lived longest as an adult.

### Cleaning and processing of address data

LexisNexis provided a set of addresses for each participant that included the street address, city, state, zip code, and start and stop dates “seen” for each address. To create continuous residential histories from the set of LexisNexis addresses, we adapted a published algorithm to clean the address data [6]. After excluding addresses with missing information, we used the USPS RDI identify and exclude business addresses. We also excluded addresses with timeframes not within or overlapping the period from 1980 through the study enrollment year and truncated address stop years at the enrollment year.

We performed several steps to reconcile incongruities in timeframes of the cleaned address data. First, to ensure the residential histories reflected time that can be linked to meaningful exposure durations relative to chronic disease outcomes, we excluded short duration addresses (≤31 days) [6]. Next, when self-reported baseline or longest adult address locations matched the LexisNexis records, we substituted the LexisNexis dates with self-reported dates of residence. Then, we sorted addresses by their start dates. When there were matching street addresses, we combined the time frames (which also reconciled duplicate addresses). When gaps or overlaps existed, we assigned the start date of the following address as the end date of the preceding address. We followed this procedure for resolving gap and overlaps in address histories given evidence that start dates in LexisNexis are more accurate than end dates [6].

### Address validation sample

Of the participants with LexisNexis residential history data, we selected 1000 women to participate in an address validation study. To ensure the sample included participants across sociodemographic groups to facilitate comparisons, we drew a weighted random sample based on baseline age and self-reported race and ethnicity: 25% non-Hispanic White (NHW) and ≤55 years, 25% NHW and >55 years, 25% non-NHW and ≤55 years, and 25% non-NHW and >55 years.

Study participants selected into the validation sample were asked to complete an address validation questionnaire. The form was personalized for each participant and included a list of their LexisNexis addresses (street name and number, city, state, zip code, and corresponding years of residence) up to their year of study enrollment. For each address, participants had the option to select “No updates” if the address was correct, or “Yes, updates” if any of the provided address information was incorrect. If participants selected “Yes, updates,” they were instructed to provide the correct address information. Separately, participants were asked to provide address information for any residences at which they lived between 1980 and their enrollment year that were not included on the form.

### Descriptive summaries and analysis

Among all participants with available residential history data, we summarized the distribution of the number of addresses per participant, duration of residence (years) each address, total years of address history, and age at the start of the earliest address overall and by sociodemographic characteristics.

To evaluate the accuracy of LexisNexis address locations among the validation sample, we calculated the proportion of address confirmed at the detailed street (street name and number), street name, zip code, city, and state level, as well as the proportion of LexisNexis addresses that were assigned to the same census tract as verified/corrected address. To evaluate the accuracy of timing, we calculated the proportion with confirmed dates of residence (start and/or stop year) and the percent of time (years) correctly covered by the LexisNexis addresses.

Address validation metrics were calculated overall and by self-reported sociodemographic characteristics collected at baseline: age (≤45, 46 to 56, 56 to 65, and >65 years), race and ethnicity [Hispanic, non-Hispanic Black, NHW, additional groups (including American Indian or Alaska Native, Asian, Native Hawaiian or other Pacific Islander, and unknown or not specified)], educational attainment (high school graduate or lower, some college, four-year degree or higher), and household income (<$50,000, $50,000–$99,999, and ≥$100,000). We also calculated proportions by address urbanicity [based on 2003 USDA Rural-Urban Continuum Codes [14]: urban/metro [1–3], urban/non-metro [4–7], or rural county [8, 9] and census region (Midwest, Northeast, South, West, Puerto Rico). To make the results representative of the overall population with address history data, we calculated a weighted mean proportion for the overall sample, age groups, and race and ethnicity groups to account for sampling proportions; for all other groups, we calculated a simple proportion. Weights were calculated as the ratio of each sampling group’s proportion among analyzed respondents to their proportion in the full cohort with address history data. To assess differences between subgroups, we obtained *p*-values from chi-square tests of independence ($$\alpha$$ = 0.05; tests for age and race/ethnicity groups were performed on weighted counts).

For addresses with a correction to location or dates of residence, we summarized the distance (km) and difference in years, respectively, between the geocoded LexisNexis and updated addresses.

## Results

### Residential history data

LexisNexis identified 47,557 (93.5%) study participants in its database and returned a total of 497,323 addresses, ranging from 1 to 20 addresses per participant. The sociodemographic characteristics of women identified in the LexisNexis database were similar to the entire cohort (Table 1). After data cleaning and processing, we generated residential histories for all 47,557 participants, with a mean of 3.5 addresses per participant [standard deviation (SD): 2.3] and a mean duration of 7.7 years (SD: 8.9) at each study address (Table 2). There was a median of 25 years [interquartile range (IQR): 21.6–30.5] of residential history, with a median age of 28 years (IQR: 23.1–33.6) at the start year of the earliest address. The number of addresses, duration lived at each address, total years of residential history, and start age of earliest address are summarized by participants sociodemographic characteristics in Table 2.

**Table 1:** Baseline characteristics of participants in the Sister Study cohort and the address verification study.

	Overall	With residential history data	Not identified in LexisNexis	Address Verification study	Completed questionnaire	Did not complete questionnaire
	*N* = 50,884	*N* = 47,557	*N* = 3327	*N* = 1000	*n* = 823	*n* = 177
Age at baseline, *n* (%)						
≤45 years	7871 (15.5%)	7715 (16.2%)	156 (4.7%)	170 (17.0%)	141 (17.1%)	29 (16.4%)
46 to 55 years	18,362 (36.1%)	17,779 (37.4%)	583 (17.5%)	330 (33.0%)	268 (32.6%)	62 (35.0%)
56 to 65 years	17,274 (33.9%)	16,070 (33.8%)	1204 (36.2%)	413 (41.3%)	349 (42.4%)	64 (36.2%)
>65 years	7377 (14.5%)	5993 (12.6%)	1384 (41.6%)	87 (8.7%)	65 (7.9%)	22 (12.4%)
*P-*value		0.0			0.16	
Race and ethnicity, *n* (%)						
Hispanic	2515 (4.9%)	2350 (4.9%)	165 (5.0%)	122 (12.2%)	94 (11.4%)	28 (15.8%)
Non-Hispanic Black	4462 (8.8%)	4227 (8.9%)	235 (7.1%)	292 (29.2%)	227 (27.6%)	65 (36.7%)
Non-Hispanic White	42,558 (83.6%)	39,731 (83.5%)	2827 (85.0%)	500 (50.0%)	433 (52.6%)	67 (37.9%)
Additional groups	1334 (2.6%)	1235 (2.6%)	99 (3.0%)	86 (8.6%)	69 (8.4%)	17 (9.6%)
Missing	15 (0.0%)	14 (0.0%)	1 (0.0%)	0 (0%)	0 (0%)	0 (0%)
*P-*value		0.0			0.0	
Educational attainment, *n* (%)						
High school graduate or less	7804 (15.3%)	7100 (14.9%)	704 (21.2%)	122 (12.2%)	90 (10.9%)	32 (18.1%)
Some college/technical school	17,180 (33.8%)	15,959 (33.6%)	1220 (36.7%)	351 (35.1%)	275 (33.4%)	76 (42.9%)
Four-year degree or more	25,883 (50.9%)	24,486 (51.5%)	1394 (41.9%)	527 (52.7%)	458 (55.7%)	69 (39.0%)
Missing	17 (0.0%)	12 (0.0%)	9 (0.3%)	0 (0%)	0 (0%)	0 (0%)
*P-*value		0.0			0.0	
Household income, *n* (%)						
Less than $50,000	13,118 (25.8%)	11,640 (24.5%)	1478 (44.4%)	266 (26.6%)	199 (24.2%)	67 (37.9%)
$50,000–$99,999	20,751 (40.8%)	19,567 (41.1%)	1184 (35.6%)	399 (39.9%)	327 (39.7%)	72 (40.7%)
$100,000 or more	17,006 (33.4%)	16,350 (34.4%)	656 (19.7%)	335 (33.5%)	297 (36.1%)	38 (21.5%)
Missing	9 (0.0%)	0 (0%)	9 (0.3%)	0 (0%)	0 (0%)	0 (0%)
*P-*value		0.0			0.0	

“Not identified in LexisNexis” includes 9 participants who withdrew from the Sister Study.

*P*-values obtained from chi-square tests of independence.

**Table 2:** Summary of LexisNexis residential history data among Sister Study participants.

	*N*	No. addresses	Duration of residence per address (years)	Total years of address history	Start age of earliest address (years)
		Mean (SD)	Mean (SD)	Median [IQR]	Median [IQR]
Overall	47,557	3.5 (2.3)	7.7 (8.9)	25.0 [21.6, 30.5]	28.0 [23.1, 33.6]
Age at baseline					
≤45 years	7715	4.8 (2.4)	4.4 (5.1)	20.8 [18.6, 23.1]	20.9 [18.9, 23.0]
45 to <55 years	17,779	3.6 (2.3)	6.9 (7.4)	24.2 [21.7, 27.5]	26.5 [23.3, 29.9]
55 to <65 years	16,070	2.9 (2.1)	9.8 (10.2)	27.7 [23.4, 32.9]	32.4 [27.5, 36.8]
≥65 years	5993	2.6 (2.0)	12.6 (13.0)	32.6 [25.4, 39.6]	36.4 [29.7, 43.8]
Race and ethnicity					
Hispanic	2350	3.2 (2.4)	7.9 (9.8)	23.9 [19.0, 30.9]	27.4 [21.8, 34.1]
Non-Hispanic Black	4227	4.1 (2.6)	6.8 (8.5)	25.6 [22.7, 31.2]	25.8 [21.1, 31.2]
Non-Hispanic White	39,731	3.4 (2.3)	7.8 (9.0)	25.0 [21.6, 30.4]	28.3 [23.4, 33.8]
Additional groups	1235	3.8 (2.5)	6.8 (8.1)	24.4 [21.1, 29.2]	27.8 [22.4, 33.1]
Educational attainment					
High school graduate or less	7100	3.1 (2.2)	9.2 (10.5)	26.2 [22.1, 33.2]	27.9 [22.8, 34.1]
Some college/technical school	15,959	3.5 (2.4)	7.7 (9.0)	25.3 [21.9, 31.0]	27.6 [22.8, 33.4]
Four-year degree or more	24,486	3.5 (2.3)	7.3 (8.4)	24.5 [21.4, 29.4]	28.3 [23.4, 33.6]
Household income					
Less than $50,000	11,640	3.4 (2.5)	8.3 (10.2)	26.2 [21.8, 33.5]	29.3 [23.7, 35.9]
$50,000-$99,999	19,567	3.4 (2.3)	7.9 (9.1)	25.1 [21.6, 30.5]	27.8 [23.0, 33.3]
$100,000 or more	16,350	3.6 (2.3)	7.0 (7.8)	24.4 [21.6, 28.6]	27.5 [22.9, 32.5]

*n* = 14 missing race/ethnicity, *n* = 12 missing education.

*SD* standard deviation, *IQR* interquartile range.

### Address validation study

A total of 823 (82.3%) participants completed the address validation questionnaire. Table 3 shows the proportion of address locations verified at various spatial levels among the entire sample and by sociodemographic and geographic characteristics. The reconstructed residence histories accurately captured most address locations (94.6% at the detailed street-level). This proportion was higher among participants older than 65 years at baseline (97.6%) and lower among addresses with a start year before 1980 (89.1%). Otherwise, proportions were similar across participant sociodemographic characteristics and geographic characteristics of addresses. For all addresses with updates to location information (*n* = 272, 6.2%), the median distance between the LexisNexis and corrected addresses was 5.4 km (IQR: 0.1–41.7).

**Table 3:** Proportion (%) of addresses in LexisNexis-derived residence histories with verified locations and timeframes.

	*N* addresses	Location						Timeframe			
		Street number	Street name	Zip code	City	State	Census tract	Start year	Stop year	Start and stop year	Percent time covered
Overall	3629	94.6	94.9	96.3	96.8	98.8	95.4	79.8	75.4	71.7	81.5
Age at baseline											
≤45 years	765	93.0	93.8	95.3	96.2	97.9	93.7	81.2	75.8	73.8	84.9
46 to 55 years	1197	93.8	94.3	96.0	96.6	99.1	93.7	80.7	76.9	73.1	82.4
56 to 65 years	1389	94.3	94.5	95.9	96.4	98.7	95.0	77.7	74.2	69.8	80.0
>65 years	278	97.6	98.1	99.3	99.4	99.9	98.1	87.9	79.5	78.1	85.4
*P*-value		0.02	0.01	0.02	0.02	0.03	0.03	0.01	0.15	0.03	0.06
Race and ethnicity^a^											
Hispanic	423	94.0	94.2	95.6	96.3	98.5	94.4	77.6	73.3	71.5	78.7
Non-Hispanic Black	1068	94.1	93.9	95.2	96.2	98.1	94.3	85.2	82.3	79.7	85.1
Non-Hispanic White	1834	94.2	95.1	96.4	96.9	98.9	95.6	79.3	74.7	70.7	81.4
Additional groups	304	94.0	94.0	96.4	97.7	99.0	93.9	79.1	78.3	74.8	74.7
*P*-value		0.23	0.21	0.22	0.12	0.09	0.28	0.00	0.00	0.00	0.01
Educational attainment											
High school graduate or less	395	92.9	93.4	95.9	95.9	99.2	94.2	81.3	78.5	76.2	86.9
Some college/technical school	1247	95.3	95.7	96.6	97.3	98.9	95.6	86.5	83.6	81.0	90.8
Four-year degree or more	1987	93.7	94.1	95.5	96.4	98.4	94.5	79.7	75.7	72.3	87.2
*P*-value		0.10	0.08	0.32	0.28	0.33	0.33	0.00	0.00	0.00	0.01
Household income											
Less than $50,000	946	95.3	95.7	97.0	97.6	99.6	95.7	84.6	82.9	79.7	90.8
$50,000–$99,999	1420	94.1	94.6	95.9	96.9	98.9	95.0	83.2	79.2	76.5	88.8
$100,000 or more	1263	93.3	93.7	95.0	95.6	97.8	94.1	79.3	75.1	71.7	86.1
*P*-value		0.14	0.12	0.06	0.04	0.00	0.23	0.00	0.00	0.00	0.00
Start year of address											
Before 1980	304	89.1	89.8	93.1	93.8	97.7	90.8	69.1	70.1	63.8	81.4
1980–1984	482	92.9	93.8	95.4	96.3	99.0	94.0	76.3	76.8	72.2	87.6
1985–1989	849	94.5	94.9	96.1	96.5	98.7	94.9	86.3	82.9	80.4	92.4
1990–1994	730	95.5	95.6	96.7	97.3	98.6	96.2	84.5	81.4	77.9	90.6
1995–1999	589	94.9	95.1	96.3	97.1	99.2	95.1	84.4	77.9	76.2	89.2
2000 and after	674	94.8	95.1	96.0	97.3	98.5	95.5	82.6	76.6	74.6	84.2
*P*-value		0.00	0.01	0.16	0.07	0.59	0.01	0.00	0.00	0.00	0.00
Urbanicity^b^											
Urban meto county	3230	94.1	94.5	95.7	96.5	98.7	94.7	82.1	78.5	75.6	88.4
Urban non-metro county	308	94.2	94.2	96.4	96.8	97.7	95.1	85.7	82.8	80.2	88.5
Rural county	46	97.8	97.8	100.0	100.0	100.0	100.0	91.3	89.1	84.8	90.9
*P*-value		0.56	0.59	0.31	0.43	0.25	0.26	0.08	0.05	0.07	0.87
Census region											
Midwest	799	94.1	94.9	96.2	96.6	98.6	95.4	81.7	79.8	75.3	88.1
Northeast	509	92.3	92.9	95.1	96.1	98.6	92.5	80.0	76.2	73.3	87.0
South	1,428	95.0	95.2	96.4	97.2	98.5	95.5	85.2	80.9	78.9	89.4
West	804	94.0	94.3	95.1	96.1	99.0	94.7	81.2	77.5	74.8	88.6
Puerto Rico	37	89.2	89.2	96.4	94.6	100.0	91.9	64.9	62.2	59.5	76.6
*P*-value		0.17	0.00	0.35	0.47	0.82	0.06	0.01	0.08	0.03	0.52

Weighted mean proportions were calculated for the following groups: overall, age at baseline, and race and ethnicity.

Except for census tract, verification at a given spatial level implies verification at all larger spatial levels.

*P*-values obtained from chi-square tests of independence (counts for age groups and race and ethnicity groups were weighted according to sampling weights).

^a^Additional groups include American Indian or Alaska Native, Asian, Native Hawaiian or other Pacific Islander, and unknown or not specified.

^b^Based on 2003 USDA Rural-Urban Continuum Codes: urban/metro [1–3], urban/non-metro [4–7], or rural county [8, 9].

*n* = 45 addresses missing urbanicity; *n* = 52 addresses missing census region.

While 71.7% of addresses had both verified start and stop dates of residence, the residential histories accurately covered 81.5% of verified/corrected address timeframes. The precent of time covered varied by race and ethnicity, educational attainment, household income, and start year of address (*P*-value < 0.01, see Table 3). A greater proportion of start dates of residence (79.8%) were verified than end dates (75.4%). For addresses with updates to dates of residence (*n* = 546, 15%), the median difference in duration of residence between LexisNexis and corrected addresses was +/- 3 years (IQR: 1–5).

On the address validation questionnaire, participants separately reported a total of 338 additional addresses at which they lived during or overlapping the period between 1980 and study enrollment that were not included in the LexisNexis residential history. For over half (58.9%) of these addresses, participants indicated that the start year of residence was before 1985, a period for which LexisNexis data is less complete.

## Discussion

Using address records from the commercial database LexisNexis, we reconstructed retrospective residential histories for 93.5% of participants in the Sister Study cohort. This effort added a median of 25 years of address history prior to study enrollment. For a subset of participants, 95% of address locations and 82% of the time spent at addresses were verified, demonstrating the accuracy of the reconstructed residential histories. The residential data produced in this analysis provides a valuable resource for future studies leveraging geospatial data to understand the health impacts of social and environmental exposures across the lifecourse.

Our results align with prior literature that finds LexisNexis records correspond well with study address locations [7–12]. Most of these studies evaluated agreement between study and LexisNexis addresses at the year of study baseline or completion of a follow-up questionnaire. For example, the overall proportion of study baseline addresses that matched LexisNexis address records was 86% in both the California Teachers Study [10] and the Los Angeles Ultrafines Study [7] and 92% in the nationwide REGARDS cohort [11]. These proportions are comparable to our findings where 95% of LexisNexis address locations were confirmed in the address validation sample. However, because of the limited extent of self-reported address data, these prior studies were unable to evaluate the ability of LexisNexis to capture all residential moves continuously across time.

In our study, we evaluated longitudinal address histories, allowing us to describe temporal mismatches across multiple addresses. We found residential histories accurately covered 82% of the time spent at of addresses, and this proportion improved after 1985 when LexisNexis data is more complete. Our results are comparable to two other studies that also evaluated address location and timing [8, 9]. Among participants in a Michigan case-control study, Jacquez et al. [9] compared recalled lifetime residential histories against LexisNexis and found that LexisNexis addresses covered 72% of the time spent at lifetime addresses. However, the authors obtained only the 3 most recent LexisNexis addresses for each individual, which limited the length of residential history available for comparison. In another analysis among 1000 participants in the NIH-AARP Diet and Health Study, Wheeler and Wang [8] found that 86% of follow-up study addresses matched LexisNexis records at the detailed street-level, and for those matched addresses, LexisNexis records covered 89% of the time spent at them. Despite differences between this analysis and our study in the age of study participants and benchmark for comparison (study addresses in the NIH-AARP cohort were identified using a combination of self-report and the USPS National Change of Address product, whereas we used participant recall to verify LexisNexis addresses), the overall findings are similar.

Prior studies have reported that for certain sociodemographic groups or geographic regions, the completeness and accuracy of LexisNexis data can vary [10–12]. We observed few significant differences in the accuracy of address locations but some differences in the accuracy of timeframes across socioeconomic groups. The mechanisms that may lead to biases in the accuracy of residence histories include historic segregation that influences contemporary housing insecurity among Black and other racially minoritized groups [15]. For groups with high socioeconomic position, it can be challenging to distinguish primary residences from simultaneously owned properties or businesses. Consistent with prior studies, Sister Study participants who were not identified in the LexisNexis database differed from the overall cohort were older, had less education and lower incomes. This likely reflects bias inherent to commercial address data whereby people with a lower socioeconomic position are less likely to engage in activities (e.g., purchasing property, voting, or registering vehicles) that generate residential information in administrative databases [15]. It is reassuring that the proportion of Sister Study participants identified in LexisNexis was high (93.5%) and consistent with other studies at the same time period [7, 11, 12], although careful attention should be paid to potential selection or information biases introduced by linking commercial address data to cohort studies, such as greater levels of exposure misclassification among groups with less accurate resident histories [16]. Additionally, our findings from the Sister Study—which has an overall higher socioeconomic position than the general US population—may not be generalizable to other cohorts with a different age distribution, sociodemographic makeup, or study period. Future studies exploring the use of LexisNexis or other sources of commercial address data should be mindful of the time period and population characteristics that may impact data completeness and accuracy.

This study contributes to the knowledge of best practices for cleaning and processing commercial address data for research use. Xu et al. [12] provided the first in-depth description of a “standard” procedure for generating residential histories using raw data from LexisNexis. We followed a similar procedure in cleaning LexisNexis address data with the additional step of integrating self-reported study addresses to help resolve timing incongruities and improve our confidence in the final residential histories. Thus, our study complements the blueprint described in Xu et al. [12] by demonstrating how additional address data sources (i.e., self-reported study addresses and RDI) can be used in generating LexisNexis residence histories.

In this study, we described the utility of LexisNexis for generating retrospective detailed residential histories in a nationwide cohort of women. Our findings confirmed that the address histories accurately represented a high proportion of locations and time frames. These results highlight the value of this address information for use in future epidemiologic studies.

## Data Availability

Requests for data, including the data and code used in this manuscript, are welcome. De-identified data are made available upon request as described on the public study website (https://sisterstudy.niehs.nih.gov/English/data-requests.htm). The data sharing policy was developed to protect the privacy of study participants and is consistent with study informed consent documents as approved by the NIEHS institutional review board.

## References

[1] Medgyesi DN, Fisher JA, Cervi MM, Weyer PJ, Patel DM, Sampson JN, et al. Impact of residential mobility on estimated environmental exposures in a prospective cohort of older women. Environ Epidemiol. 2020;4:e110. 33154988 10.1097/EE9.0000000000000110 PMC7595244

[2] Ling C, Heck JE, Cockburn M, Liew Z, Marcotte E, Ritz B. Residential mobility in early childhood and the impact on misclassification in pesticide exposures. Environ Res. 2019;173:212–20. 30928851 10.1016/j.envres.2019.03.039 PMC6553500

[3] Christian WJ, Walker CJ, Huang B, Levy JE, Durbin E, Arnold S. Using residential histories in case-control analysis of lung cancer and mountaintop removal coal mining in Central Appalachia. Spatial Spatio-temporal Epidemiol. 2020;35:100364. 10.1016/j.sste.2020.100364 PMC7609974 33138948

[4] Liu B, Niu L, Lee FF. Utilizing residential histories to assess environmental exposure and socioeconomic status over the life course among mesothelioma patients. J Thorac Dis. 2023;15:6126–39. 38090310 10.21037/jtd-23-533 PMC10713296

[5] Semmens EO, Leary CS, Fitzpatrick AL, Ilango SD, Park C, Adam CE, et al. Air pollution and dementia in older adults in the Ginkgo Evaluation of Memory Study. Alzheimer’s Dement. 2023;19:549–59. 35436383 10.1002/alz.12654 PMC9576823

[6] Stinchcomb DG, Roeser A. NCI/SEER residential history project. Rockville, MD; Westat, Inc. 2016.

[7] Medgyesi DN, Fisher JA, Flory AR, Hayes RB, Thurston GD, Liao LM, et al. Evaluation of a commercial database to estimate residence histories in the Los Angeles ultrafines study. Environ Res. 2021;197:110986. 33689822 10.1016/j.envres.2021.110986 PMC8187285

[8] Wheeler DC, Wang A. Assessment of residential history generation using a public-record database. Int J Environ Res Public Health. 2015;12:11670–82. 26393626 10.3390/ijerph120911670 PMC4586699

[9] Jacquez GM, Slotnick MJ, Meliker JR, AvRuskin G, Copeland G, Nriagu J. Accuracy of commercially available residential histories for epidemiologic studies. Am J Epidemiol. 2011;173:236–43. 21084554 10.1093/aje/kwq350 PMC3025779

[10] Hurley S, Hertz A, Nelson DO, Layefsky M, Von Behren J, Bernstein L, et al. Tracing a path to the past: exploring the use of commercial credit reporting data to construct residential histories for epidemiologic studies of environmental exposures. Am J Epidemiol. 2017;185:238–46. 28073765 10.1093/aje/kww108 PMC5860230

[11] Brooks MS, Bennett A, Lovasi GS, Hurvitz PM, Colabianchi N, Howard VJ, et al. Matching participant address with public records database in a US national longitudinal cohort study. SSM Popul Health. 2021;15:100887. 34401464 10.1016/j.ssmph.2021.100887 PMC8358447

[12] Xu W, Agnew M, Kamis C, Schultz A, Salas S, Malecki K, et al. Constructing residential histories in a general population-based representative sample. Am J Epidemiol. 2024;193:348–59. 37715463 10.1093/aje/kwad188 PMC10840075

[13] Sandler DP, Hodgson ME, Deming-Halverson SL, Juras PS, D’Aloisio AA, Suarez LM, et al. The Sister Study cohort: baseline methods and participant characteristics. Environ Health Perspect. 2017;125:127003. 29373861 10.1289/EHP1923 PMC5963586

[14] U.S. Department of Agriculture ERS. Rural-Urban Continuum Codes. https://www.ers.usda.gov/data-products/rural-urban-continuum-codes.

[15] Sims KD, Glymour MM, Ncube CN, Willis MD. Invited commentary: Improving spatial exposure data for everyone-life-course social context and ascertaining residential history. Am J Epidemiol. 2025;194:573–7. 10.1093/aje/kwae244 PMC11879526 39098825

[16] Freeman VL, Boylan EE, Tilahun NY, Basu S, Kwan M-P. Sources of selection and information biases when using commercial database–derived residential histories for cancer research. Ann Epidemiol. 2020;51:35–40.e1. 10.1016/j.annepidem.2020.07.010 32711052

